# Sparse clusterability: testing for cluster structure in high dimensions

**DOI:** 10.1186/s12859-023-05210-6

**Published:** 2023-03-31

**Authors:** Jose Laborde, Paul A. Stewart, Zhihua Chen, Yian A. Chen, Naomi C. Brownstein

**Affiliations:** 1 Department of Biostatistics and Bioinformatics, Moffitt Cancer Center, Tampa, FL USA; 2 Department of Oncologic Sciences, University of South Florida, Tampa, FL USA; 3 Department of Public Health Sciences, Medical University of South Carolina, Charleston, SC USA

**Keywords:** Cluster analysis, Cluster tendency, Clustering, Big data, Dimension reduction, Principal component analysis, Distance metrics, Multimodality testing, Sparsity

## Abstract

**Background:**

Cluster analysis is utilized frequently in scientific theory and applications to separate data into groups. A key assumption in many clustering algorithms is that the data was generated from a population consisting of multiple distinct clusters. Clusterability testing allows users to question the inherent assumption of latent cluster structure, a theoretical requirement for meaningful results in cluster analysis.

**Results:**

This paper proposes methods for clusterability testing designed for high-dimensional data by utilizing sparse principal component analysis. Type I error and power of the clusterability tests are evaluated using simulated data with different types of cluster structure in high dimensions. Empirical performance of the new methods is evaluated and compared with prior methods on gene expression, microarray, and shotgun proteomics data. Our methods had reasonably low Type I error and maintained power for many datasets with a variety of structures and dimensions. Cluster structure was not detectable in other datasets with spatially close clusters.

**Conclusion:**

This is the first analysis of clusterability testing on both simulated and real-world high-dimensional data.

## Background

Cluster analysis theory, implementation, and applications are popular research topics in fields from computer science to statistics to oncology, biology, marketing, and more. Yet, most clustering algorithms output a set of clusters regardless of whether natural grouping is present in the data. Clusterability tests are designed to address this problem by capturing underlying cluster structure-or a lack thereof. Widespread use of valid clusterability tests may help orient researchers away from conducting cluster analysis when it lacks practical meaning.

A recent analysis of clusterability methods [1] identifies clusterability tests with adequate Type I error control and explores their empirical performance in identifying clusterable and unclusterable data in low dimensions [2]. In brief, clusterability tests that reduce the data to a single dimension and then implement a multimodality test perform well empirically on low-dimensional data consisting of a small number of clusters [1]. These tests have low Type I error rates—erroneously classifying datasets without clusters as having clusters—and high power—correctly identifying the presence of clusters when they exist.

Dimension reduction methods include principal component analysis (PCA) and distance metrics. An example of a distance metric is the Euclidean distance between two pairs of points in the data. Euclidean distances were used for dimension reduction because of their use as inputs to some clustering algorithms. PCA was previously chosen for dimension reduction because it maximizes the variance explained out of possible linear projections of the data from high dimensions into a one-dimensional space. By explaining the maximum variance in the original data, the first principal component has been shown to capture modes indicating the presence of clusters [1].

Existing clusterability methods have not been designed for high dimensional data. Clusterability tests depend on a unidimensional reduction, which may result in a loss of information that is more apparent for data with higher dimensions. Methods that project the data to its first principal component, a linear combination of the features in the data, may fail to separate clusters that do not fall in the directions of maximal variance [3], become more computationally intensive to compute, and can lack interpretability when the number of features is large [4]. Sparse PCA, which has not been explored prior to the writing of this paper, could be chosen as an alternative projection. The presence of zeros for many components maximizes interpretability of the sparse PCA components while maintaining a reasonable projection [4]. Distance reduction methods run in quadratic or higher time based on the number of observations [1], potentially rendering them computationally challenging for large “omics” datasets. Distance-based methods have also been shown to have poor performance for imaging datasets [5].

Moreover, prior comparisons of clusterability methods have featured data with a small number of features. Simulations were examined less than 50 features, and empirical applications featured small famous or toy datasets within the R datasets package [1, 2, 6]. Another clusterability metric designed for imaging data was tested on empirical samples with less than 300 features and simulations with 400 features [5]. Methods tested on data with a larger number of features are lacking. Feasibility and generalizability of clusterability tests for “omics” data are unclear.

This paper presents a pair of clusterability tests designed for data with a large number of observations, features, or both. We evaluate the performance of the proposed clusterability tests on simulated and empirical biomedical data with high dimensions and compare with previously developed tests. The goals of the paper are to (1) create clusterability tests for high dimensional data, and (2) evaluate the performance of clusterability tests in high dimensional simulated and real-world data. The Methods section details the proposed and existing clusterability tests, including the required dimension reduction techniques and multimodality tests. The Results section features simulations evaluating Type I error and power of the tests. The Results section also demonstrates applications of the methods to multiple large cancer-omics datasets. Finally, we conclude with a discussion of the findings and future work.

## Results

### Simulations

We simulated datasets of varying dimensionality with and without cluster structure, and we tested the agreement of the clusterability tests with the expected conclusion associated with each dataset. Type I error, power, and computational time are provided.

#### Simulation setup details

**Fig. 1:**
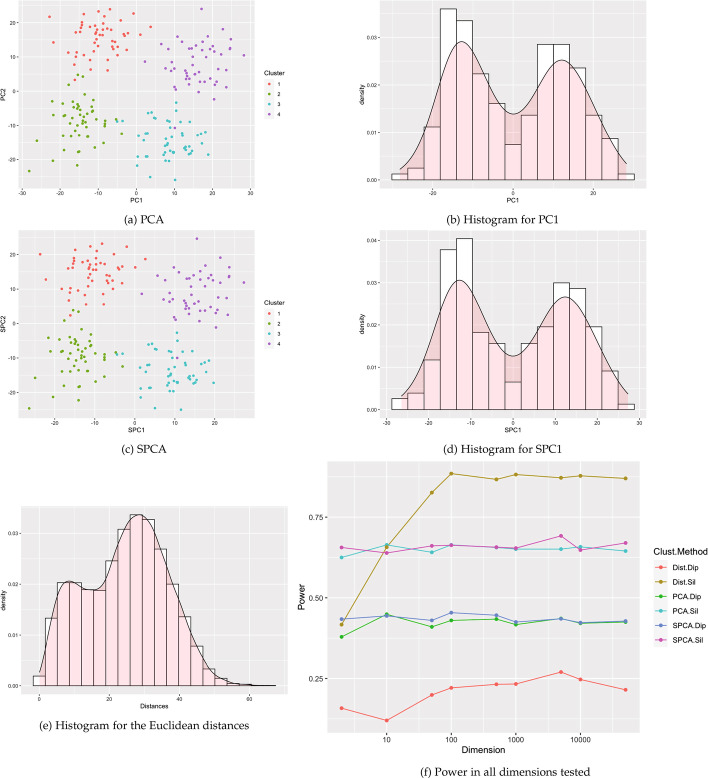
Example visualizations based 4 Gaussian clusters with equal variances (Case 2). **a**–**e** are example visuals based on $p=500$ dimensions. **f** shows power estimates in dimensions from $p=2$ to 50,000, using 1000 simulated data sets of $n=200$ observations for each dimension

**Fig. 2:**
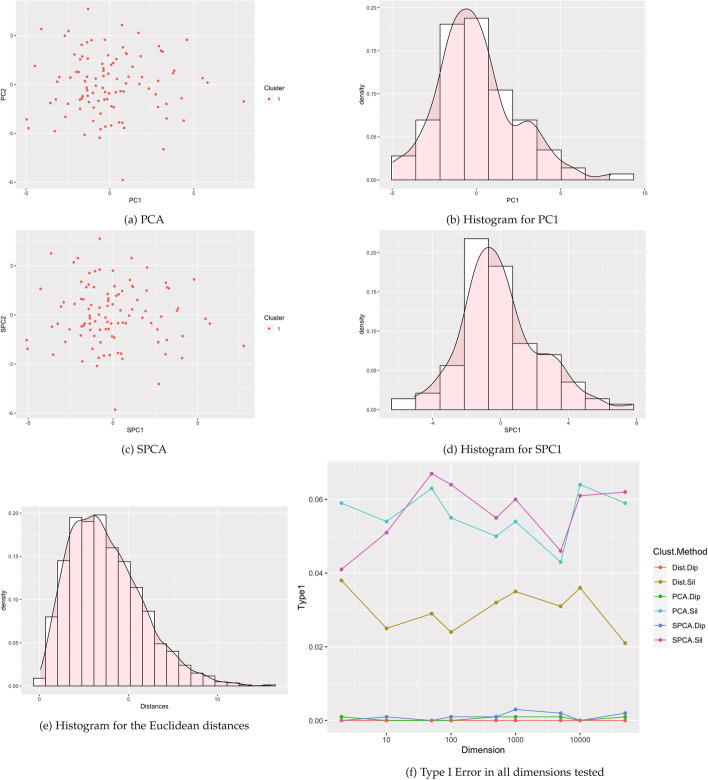
Visualizations based on a single cluster (Case 1). **a**–**e** are example visuals based on $p=500$ dimensions. **f** shows Type I error estimates in dimensions from $p = 2$ to 50,000, using 1000 simulated data sets for each dimension

Clusters were created using the clusterlab R package [7].^1^ Clusterlab generates clusters of a user-provided dimension by a linear projection of two-dimensional Gaussian principal components into the desired higher-dimensional space. The clusterlab manual highlights twelve example two-dimensional structures to project into higher dimension. In this paper, we conduct simulations based on the first ten examples. These example cases vary from a single cluster to multiple clusters with different degree of separation, variances, and outliers. Visualizations of the two-dimensional projections of the examples are included in Figs. 1 and 2 and Figs. 9, 10, 11, 12, 13, 14, 15, and 16 in the appendix. All scenarios were tested on the following dimensions: $p=2, 10, 50, 100, 500, 1000, 5000, 10000, 50000$.

**Fig. 3:**
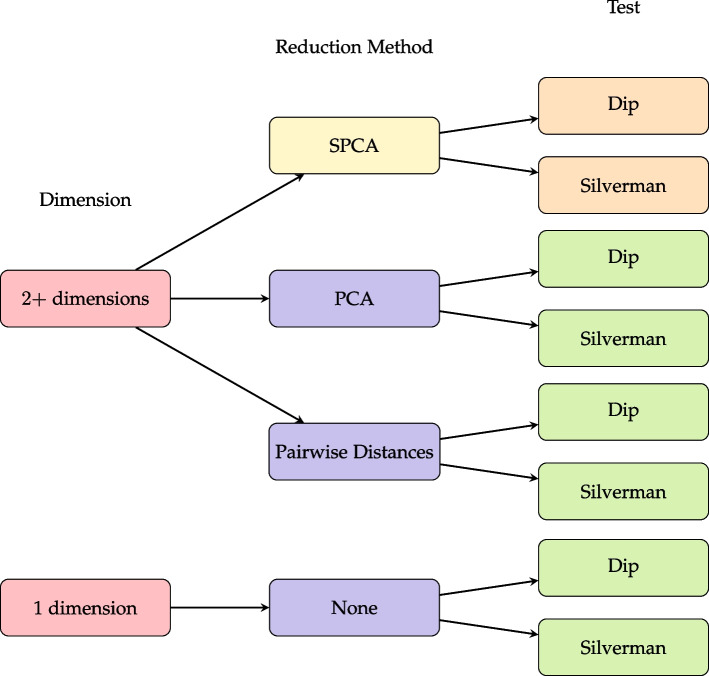
Flowchart for options of data reduction methods and multimodality tests. New methods proposed in this paper are denoted by the yellow bubble for SPCA as a new dimension reduction method, combined with each of two available multimodality tests (dip and Silverman) shown with orange bubbles

After generating each dataset using clusterlab, we conduct the clusterability tests. To implement our newly proposed methods to reduce the data with SPCA, we generated the first sparse principal component using *sparsepca* from CRAN [8], which provides efficient routines to compute SPCA via variable projection [9]. For prior clusterability tests described in the Methods for Clusterability Analysis section and Fig. 3, we use the *clusterability* package available in CRAN [10]. The clusterability package can directly perform the PCA decomposition, calculate distance matrices, and run each multimodality test. For each clusterability test, we recorded the p-value and execution time (including the time for decomposition/distance and multimodality testing).

Each type of simulation is repeated 1000 times. We report the proportion of simulations for which the p-value is below 0.05. Type 1 error is estimated in each dimension with $n=100$ vectors generated according to the parameters described in Case 1 in the clusterlab vignette [7] For each dimension ranging from *p*=2 to 50,000, clusterability tests are performed and the p-values extracted. The reported proportion of p-values below 0.05 can be interpreted as an estimate of Type 1 error, because we know a priori that the data was generated from only one cluster.

To estimate power, multiple clusters are generated with 50 vectors in each cluster (i.e. $n=50c$, where *c* is the number of clusters). This setup is repeated for each of the nine scenarios containing multiple clusters (Cases 2 thrugh 10) that we examined from the clusterlab vignette. In this case, the reported proportion can be interpreted as an estimate of power to detect the generated cluster structure, because we know a priori that there data was generated from multiple clusters. Although arithmetically the same proportions are used for Type 1 error and power, the interpretations differ. For power, the null hypothesis that the data was generated from a single cluster is false, while in the previous case for Type 1 error, the null hypothesis is true.

#### Type I error results

Figure 2 shows Type 1 error estimates for each combination of dimension and type of test performed. Our proposed method utilizing SPCA with Silverman’s critical bandwidth test had type 1 error estimates close to the nominal level and close to the corresponding test with standard PCA. For the clusterability tests invoking the dip test, type 1 error rates were near zero, much less than the nominal level. The finding that the dip test is conservative in high dimensions is consistent with simulations for smaller datasets [1] and the known and needed but not yet implemented correction factor for the dip test [11]. In brief, all tests were valid with type 1 error rates close to or below the nominal level.

#### Power results

Figure 1 and Figs. 9, 10, 11, 12, 13, 14, 15, and 16 show power estimates for each scenario. In most cases, methods utilizing Silverman were more powerful than methods utilizing dip. In just two simulations (Case 3 and Case 7) where power for all methods exceeded 90%, this relationship flipped with dip overpowering Silverman counterparts for PCA and SPCA in Case 3 and distances in Case 7. For ten or more dimensions, Silverman with distances met or exceeded power for Silverman with SPCA or PCA in all but two simulations (Case 7 and 8), also with power over 90% for these methods. Overall, across the simulations, for at least 10 dimensions, the method utilizing Silverman on the set of distances tended to display the highest power. Meanwhile, one of our proposed new methods, SPCA Silverman, was among the most powerful methods. The corresponding prior method, PCA Silverman, exhibited similar performance.

Some results varied by number and orientation of clusters. For example, power for SPCA and PCA based methods remained relatively stable with dimension in 7 of the 9 cases, with variation only in cases 4 and 5. Specific details for each simulation (Cases 2–10) follow in the Appendix (Table 1).

#### Computational time

Table 2 contains the median computer time for each scenario. The Silverman test takes longer to compute than the dip test, as expected, because it is based on the bootstrap [12]. SPCA takes longer than PCA in either the Dip or Silverman case. Because the all-against-all distance matrix calculation is exhaustive, the distance reduction method was slower than the PCA reduction method in most cases. The method utilizing both distance reduction and the Silverman test takes the longest of all in most low to medium dimensions. However, SPCA based methods, regardless of the multimodality test, are the most computationally intensive at very high dimensions ($\ge 1000$ seconds, or over 16 min, in most simulations).

**Table 1:** Clusterability test results for omics data dimensions of the datasets are denoted by *n* (sample size) and *p* (number of features)

				Dip		Silverman		
Dataset	*n*	*p*	c	Reduction	P-value	Time (s)	P-value	Time (s)
Single Cell RNA-seq	7856	16845	8	SPCA	0.000000***	5210.726	0.000000***	5217.491
				PCA	0.000000***	5546.349	0.000000***	5566.507
				Distance	0.919000	9482.946	0.000000***	34418.848
Pan-cancer RNA-seq	801	20264	5	SPCA	0.000001***	413.708	0.000000***	416.862
				PCA	0.051200*	55.887	0.000000***	57.288
				Distance	1.000000	148.025	0.575000	411.212
Pan-lung microarray	304	54675	10	SPCA	0.991000	484.492	0.012300**	481.153
				PCA	0.989000	32.159	0.000566***	32.862
				Distance	0.999000	75.539	0.410000	112.489
SCC Shotgun proteomics	108	3280	3	SPCA	0.308000	11.960	0.216000	13.265
				PCA	0.849000	0.434	0.410000	2.195
				Distance	0.995000	0.473	0.222000	5.931
Glioblastoma RNA-seq	50	1750	4	SPCA	0.516000	2.731	0.291000	4.398
				PCA	0.590000	0.069	0.343000	1.559
				Distance	0.862000	0.071	0.884000	2.511

The number of clusters labeled in the dataset is denoted by *c*

Asterisks are used to denote p-values within specified ranges. *** denotes $P<0.01$. ** denotes $0.01\le P<0.05$ * denotes $0.05\le P<0.1$

Lack of asterisks denotes $P\ge 0.1$, indicating that the test fails to detect cluster structure in that dataset

**Table 2:** Median computer time in seconds

Case	Dimension	PCA.Dip	PCA.Sil	SPCA.Dip	SPCA.Sil	Dist.Dip	Dist.Sil
01	2	0.00	1.90	0.15	2.10	0.01	6.40
01	10	0.01	2.00	0.30	2.20	0.01	6.50
01	50	0.01	2.00	0.64	2.60	0.01	6.60
01	100	0.02	2.00	1.50	3.40	0.01	6.60
01	500	0.06	2.10	6.90	8.80	0.04	6.70
01	1000	0.12	2.20	14.00	16.00	0.08	7.00
01	5000	0.53	2.70	70.00	72.00	0.49	7.40
01	10000	1.30	3.70	161.00	164.00	1.10	8.40
01	50000	8.20	10.80	808.00	811.00	16.50	24.20
02	2	0.00	2.10	0.15	2.20	0.01	19.00
02	10	0.01	2.00	0.29	2.30	0.01	18.90
02	50	0.01	2.10	0.62	2.70	0.03	19.30
02	100	0.03	2.10	1.50	3.60	0.04	19.00
02	500	0.17	2.30	13.90	16.00	0.16	19.90
02	1000	0.31	2.50	27.00	29.00	0.34	20.00
02	5000	1.66	4.00	145.00	148.00	2.15	22.10
02	10000	4.20	6.80	302.00	305.00	4.60	25.60
02	50000	24.10	26.80	1529.00	1532.00	66.20	87.00
03	2	0.00	2.00	0.14	2.20	0.01	18.90
03	10	0.01	2.00	0.30	2.30	0.01	18.90
03	50	0.01	2.10	0.62	2.70	0.03	19.10
03	100	0.03	2.10	1.50	3.50	0.04	19.10
03	500	0.17	2.30	12.90	15.00	0.16	19.90
03	1000	0.30	2.50	26.00	28.00	0.34	20.00
03	5000	1.67	4.10	146.00	148.00	2.14	22.30
03	10000	4.20	6.90	303.00	305.00	4.60	25.50
03	50000	24.00	26.70	1535.00	1538.00	66.90	88.20
04	2	0.00	2.00	0.15	2.20	0.01	18.90
04	10	0.01	2.00	0.29	2.30	0.01	18.80
04	50	0.01	2.10	0.61	2.70	0.03	18.90
04	100	0.03	2.10	1.40	3.50	0.04	19.40
04	500	0.17	2.30	12.80	14.80	0.16	19.80
04	1000	0.30	2.50	25.00	27.00	0.34	20.00
04	5000	1.63	4.00	146.00	148.00	2.12	22.20
04	10000	4.20	6.80	306.00	308.00	4.60	25.40
04	50000	23.90	26.60	1533.00	1536.00	66.90	87.80
05	2	0.00	2.00	0.15	2.20	0.01	13.30
05	10	0.01	2.00	0.30	2.30	0.01	13.00
05	50	0.01	2.00	0.62	2.60	0.02	13.10
05	100	0.02	2.00	1.40	3.40	0.03	13.40
05	500	0.12	2.20	10.60	12.60	0.11	13.80
05	1000	0.23	2.30	21.00	23.00	0.22	14.00
05	5000	1.13	3.50	117.00	120.00	1.62	15.90
05	10000	3.10	5.60	261.00	263.00	3.60	18.30
05	50000	17.80	20.40	1295.00	1298.00	42.10	56.90
06	2	0.00	2.10	0.15	2.30	0.02	27.10
06	10	0.01	2.00	0.29	2.40	0.02	26.90
06	50	0.01	2.10	0.61	2.70	0.04	27.10
06	100	0.03	2.10	1.40	3.50	0.06	27.30
06	500	0.24	2.40	15.70	17.90	0.26	28.00
06	1000	0.44	2.60	31.00	34.00	0.66	28.00
06	5000	2.44	5.00	186.00	189.00	3.80	32.90
06	10000	5.80	8.60	377.00	379.00	8.20	37.70
06	50000	33.60	36.50	1887.00	1890.00	115.90	145.80
07	2	0.01	2.20	0.14	2.50	0.06	109.20
07	10	0.01	2.30	0.30	2.60	0.08	109.10
07	50	0.02	2.40	0.62	2.90	0.14	111.20
07	100	0.05	2.40	1.40	3.70	0.23	111.70
07	500	0.68	3.10	33.20	35.50	1.28	114.10
07	1000	1.59	4.00	65.00	67.00	3.26	118.00
07	5000	7.97	10.70	351.00	354.00	17.81	133.00
07	10000	17.10	20.30	725.00	728.00	39.30	155.90
07	50000	108.10	110.90	9616.00	9619.00	543.70	663.20
08	2	0.01	2.20	0.14	2.40	0.03	53.60
08	10	0.01	2.20	0.30	2.50	0.04	54.10
08	50	0.02	2.20	0.60	2.80	0.07	54.70
08	100	0.04	2.30	1.40	3.70	0.11	54.50
08	500	0.37	2.60	22.40	24.60	0.49	55.90
08	1000	0.78	3.20	44.00	47.00	1.40	58.00
08	5000	4.38	7.10	250.00	253.00	8.60	65.60
08	10000	9.60	12.40	496.00	499.00	18.90	77.80
08	50000	1.30	1.40	54.00	54.00	6.60	7.60
09	2	0.01	2.20	0.15	2.40	0.03	54.60
09	10	0.01	2.10	0.29	2.50	0.04	54.00
09	50	0.02	2.20	0.61	2.80	0.07	55.30
09	100	0.04	2.30	1.40	3.70	0.11	54.70
09	500	0.38	2.60	22.20	24.50	0.50	55.80
09	1000	0.79	3.20	45.00	48.00	1.42	59.00
09	5000	4.36	7.00	251.00	254.00	8.50	65.90
09	10000	9.70	12.50	499.00	501.00	18.80	77.20
09	50000	1.20	1.30	51.00	51.00	5.60	6.70
10	2	0.01	2.20	0.15	2.40	0.03	54.00
10	10	0.01	2.20	0.29	2.50	0.04	53.80
10	50	0.02	2.20	0.61	2.80	0.07	54.70
10	100	0.04	2.30	1.40	3.70	0.11	54.80
10	500	0.37	2.70	22.60	24.80	0.49	55.80
10	1000	0.78	3.20	44.00	47.00	1.39	58.00
10	5000	4.41	7.10	250.00	252.00	8.63	66.40
10	10000	9.80	12.70	498.00	501.00	18.80	76.80
10	50000	1.20	1.30	51.00	51.00	5.80	6.90

### Clusterability testing applied to omics data

**Fig. 4:**
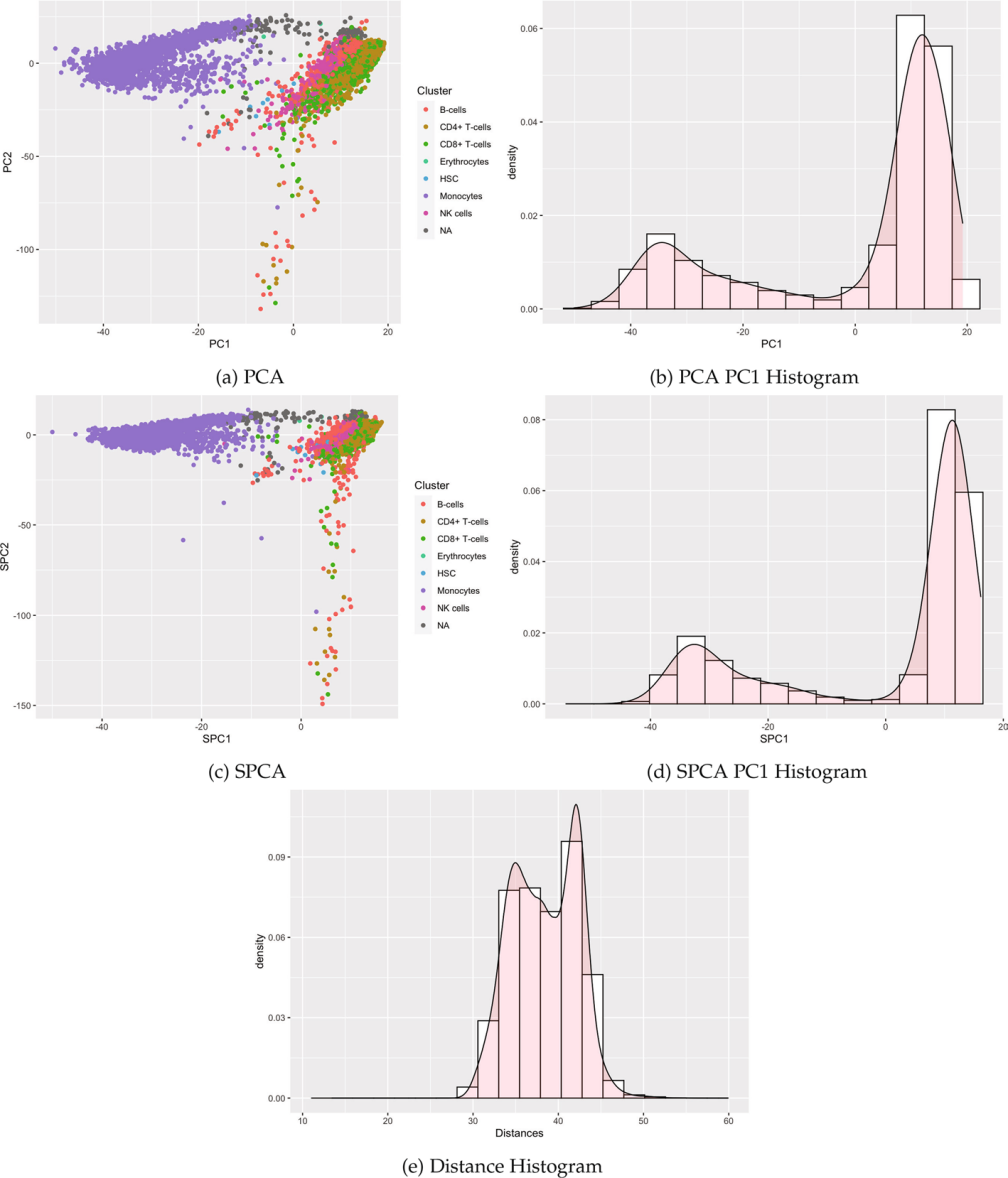
Single cell RNA-seq

**Fig. 5:**
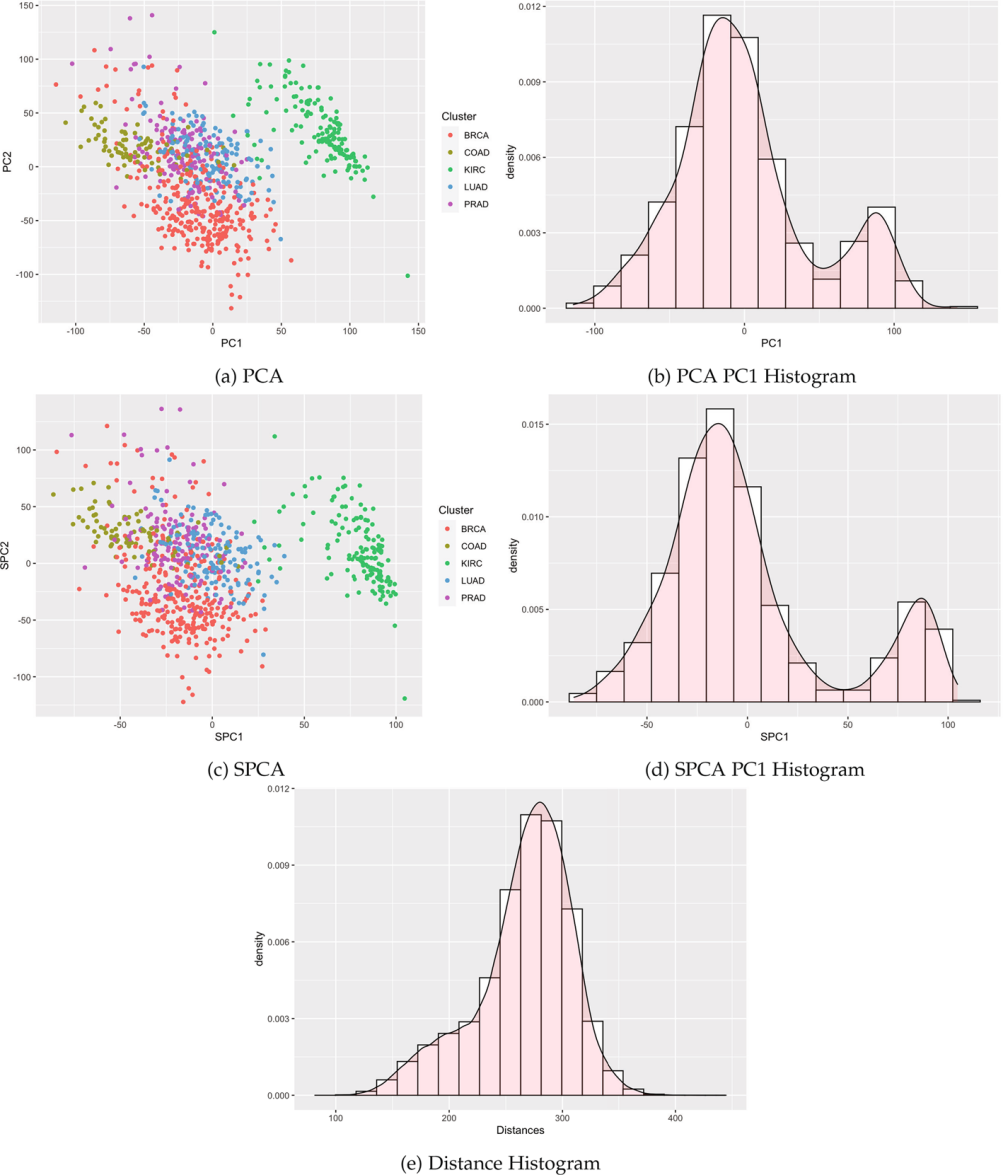
Pan-cancer RNA-seq

**Fig. 6:**
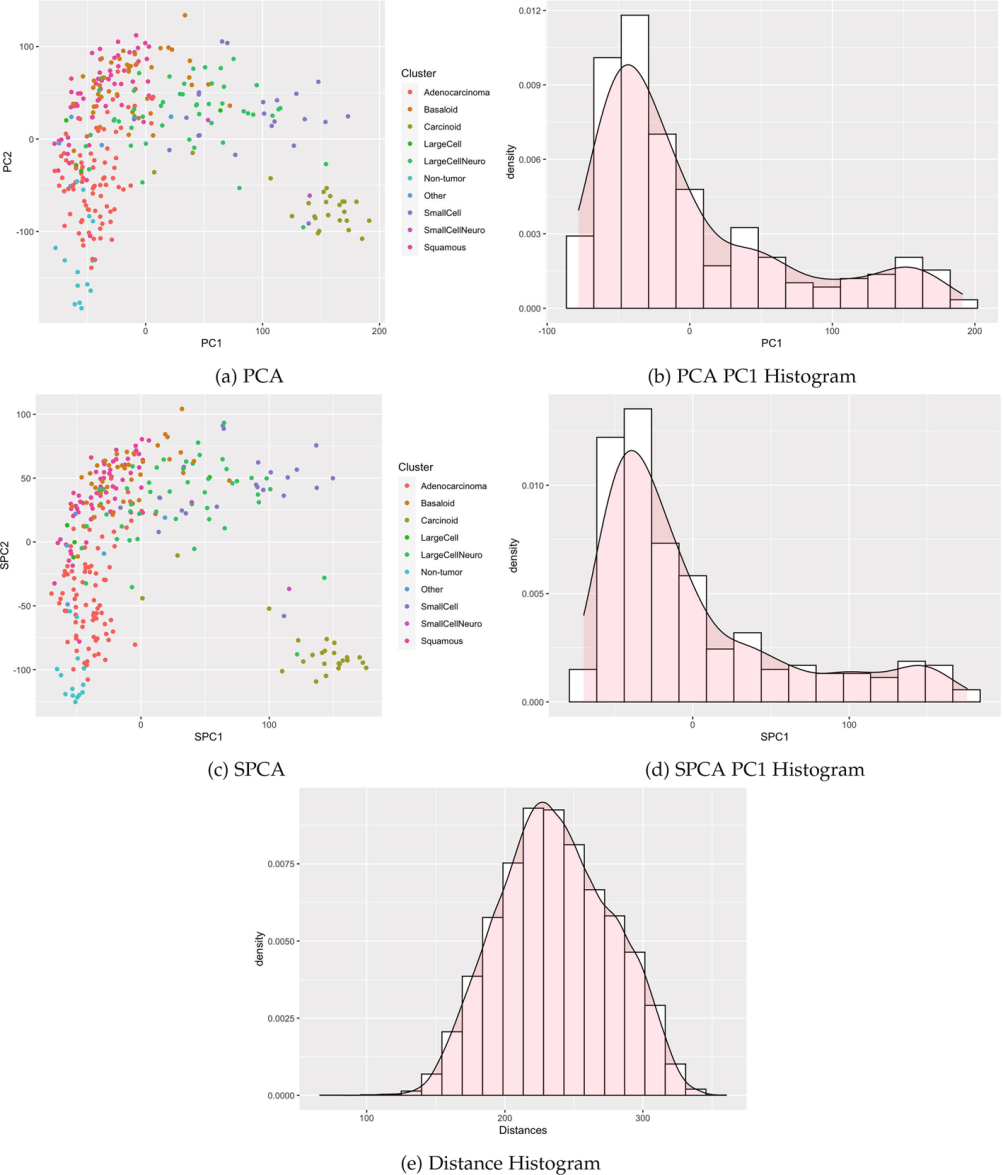
Pan-lung cancer microarray

We identified publicly available expression datasets with qualitatively different clustering patterns to examine the empirical performance of clusterability tests on a variety of data types. These examples span cluster structure detectable by all tests, structure handled differently by the tests, and clusters undetectable by each test (Figs. 4, 5, and 6).

**Fig. 7:**
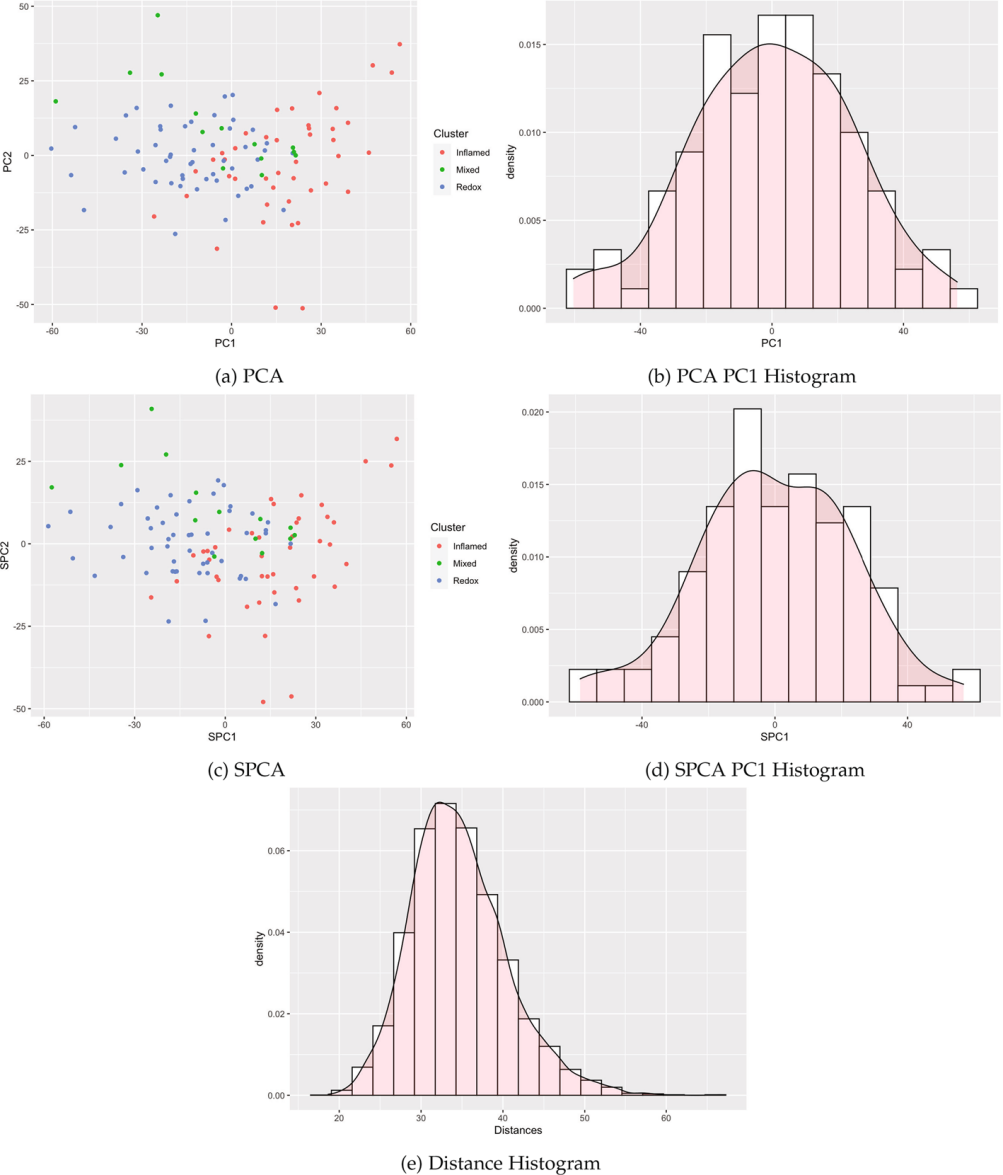
Squamous cell lung cancer proteomics

**Fig. 8:**
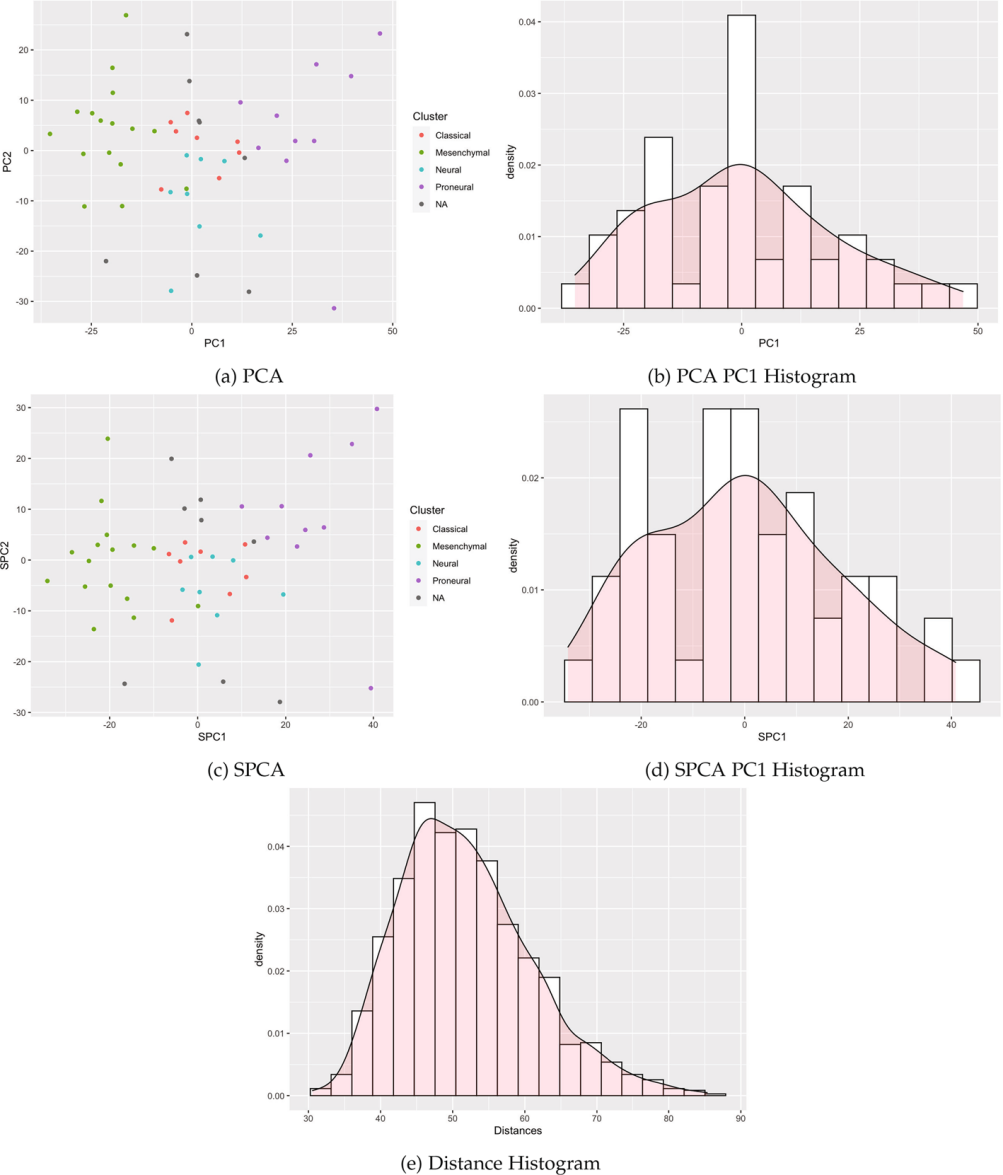
Glioblastoma RNA-seq

Table 1 summarizes results, with performance varying by method and case. Specifically, our proposed methods reducing the dimension using SPCA (and previous methods with PCA) detected the known cluster structure in some datasets with sufficient separation. Correspondingly, modes were visible in the histograms of SPC1 and PC1 (Figs. 4 and 5). All methods failed to detect structure in other datasets for which clusters were visibly intermingled (Figs. 7 and 8). By contrast, distance-based methods failed to detect cluster structure in all datasets except for the single cell data, for which a second mode was visible, and the Silverman test detected cluster structure. Other histograms of distances appeared unimodal. Consistent with the simulations, methods utilizing Silverman considered more datasets as clusterable than the applying the dip test to the same reduction method. The divergence of the tests is especially apparent in Fig. 6, where the histograms display long tails with smaller modes.

Computational time is reported in Table 1. In the single cell analysis, SPCA was the fastest method. For all other datasets, SPCA took longer than all other methods.

#### Peripheral blood mononuclear cell RNA-seq

We analyzed transcript counts from a single cell RNA sequencing experiment [13]—a 7856 cell x 16845 gene expression profile [14] of peripheral blood mononuclear cells from a healthy donor. The sample is well-characterized and known to contain various types of immune cells present in the human blood, each with a distinct RNA expression pattern [13]. The scRNA-seq dataset has previously been processed and analyzed using ISCVA [15], which is available as an online tool (http://iscva.moffitt.org/).

Visualizations of the reduced data are shown in Fig. 4. The colors show different cell types, for example with the monocytes largely clustered away from the B-cells. Distinct modes in the histograms are clearly apparent in the first principal component and sparse principal component, while the distance histogram appears more unimodal. All tests besides Hartigan’s dip test on the Euclidean distance matrix were highly significant (Table 1), indicating clearly detectable cluster structure.

**Fig. 9:**
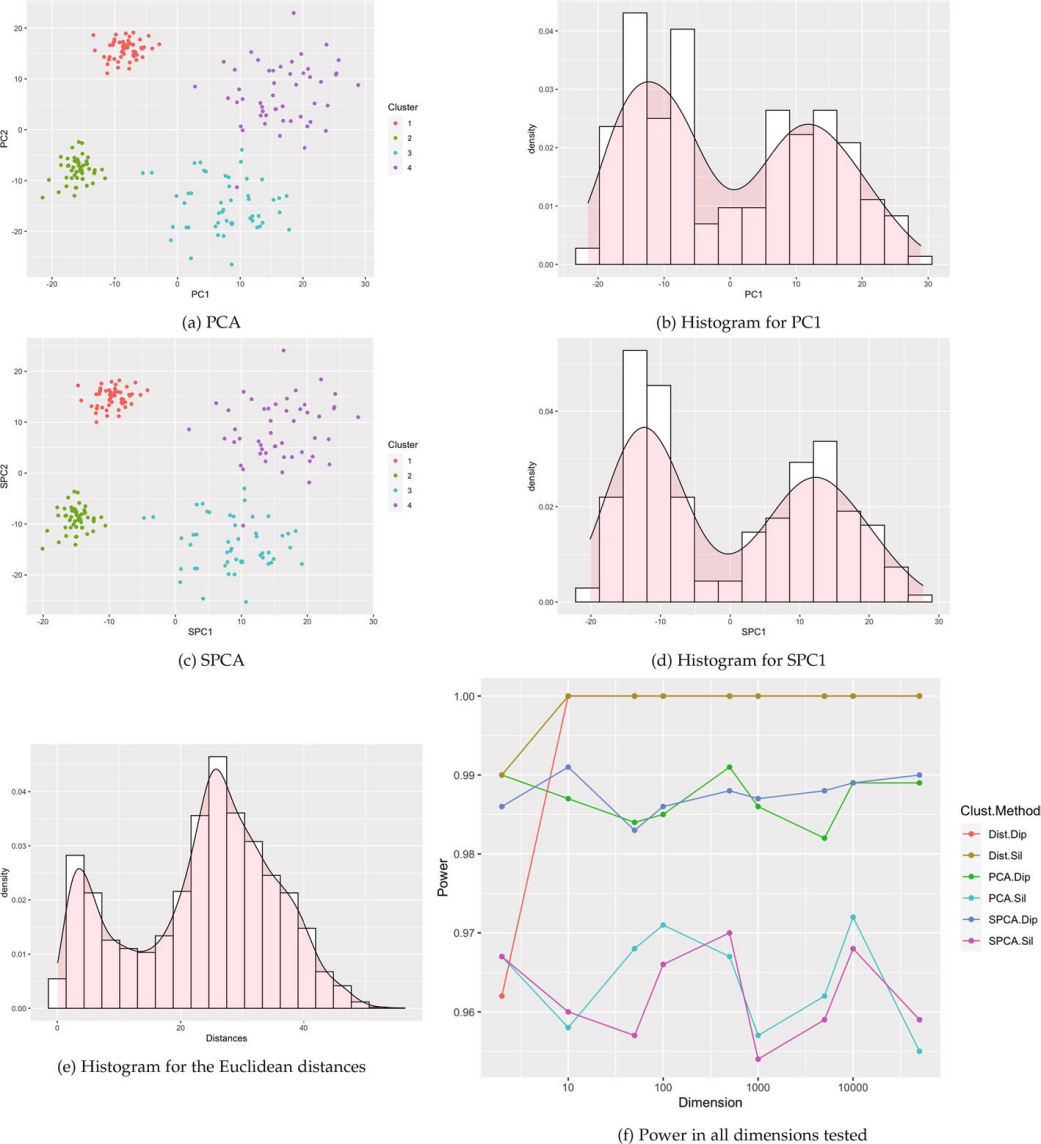
Visualizations for example Case 3: data set generated with 4 Gaussian clusters where 2 clusters have different variances to the other 2. All visualizations represent the use of a single example simulation in dimension $p=500$ except for Power which is measured in dimensions from $p=2$ to 50 K via estimation based off 1000 simulated data sets on each dimension

**Fig. 10:**
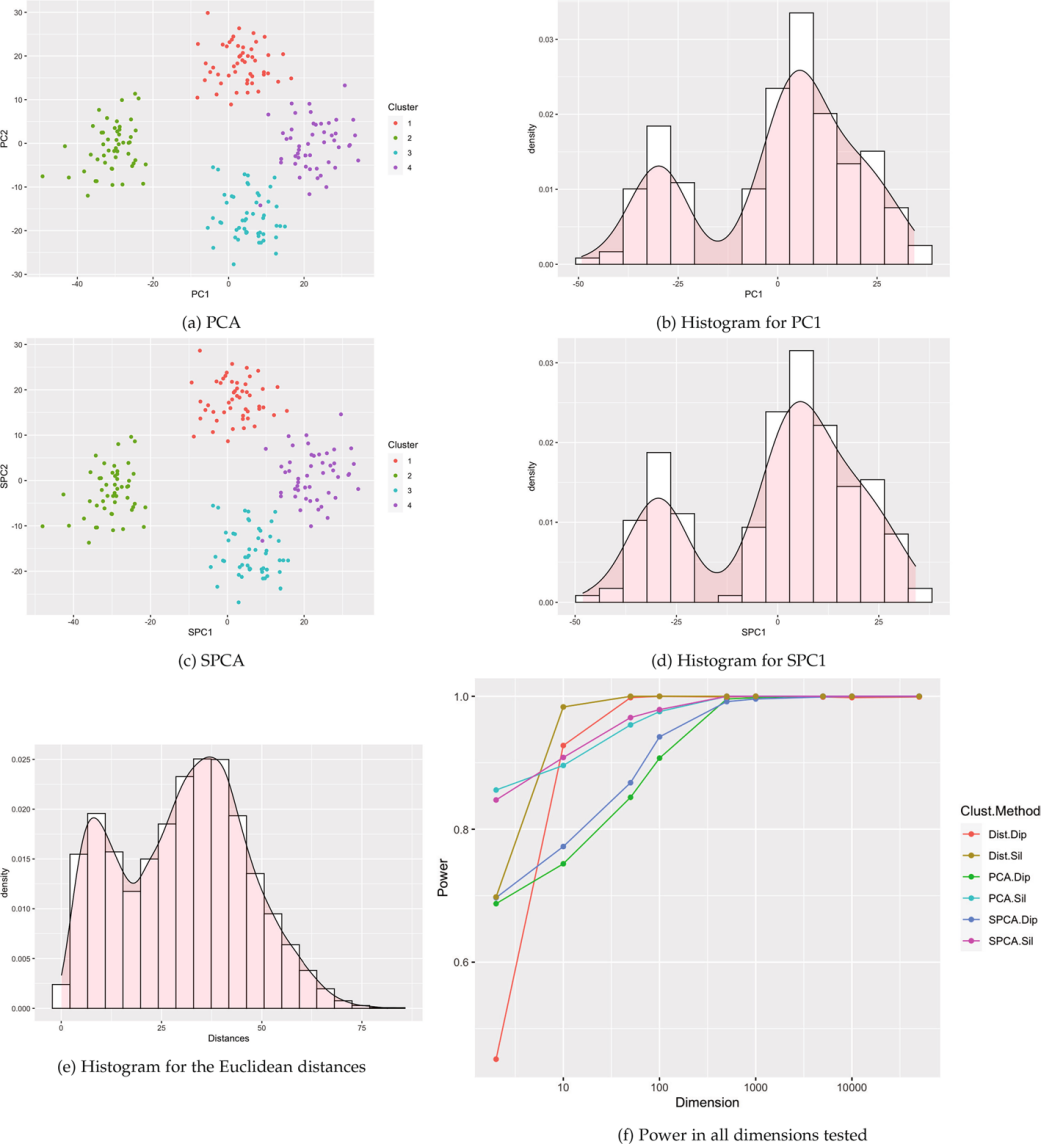
Visualizations for example Case 4: 4 Gaussian clusters with one cluster pushed to the outside. All visualizations represent the use of a single example simulation in dimension $p=500$ except for Power which is measured in dimensions from $p=2$ to 50 K via estimation based off 1000 simulated data sets on each dimension

**Fig. 11:**
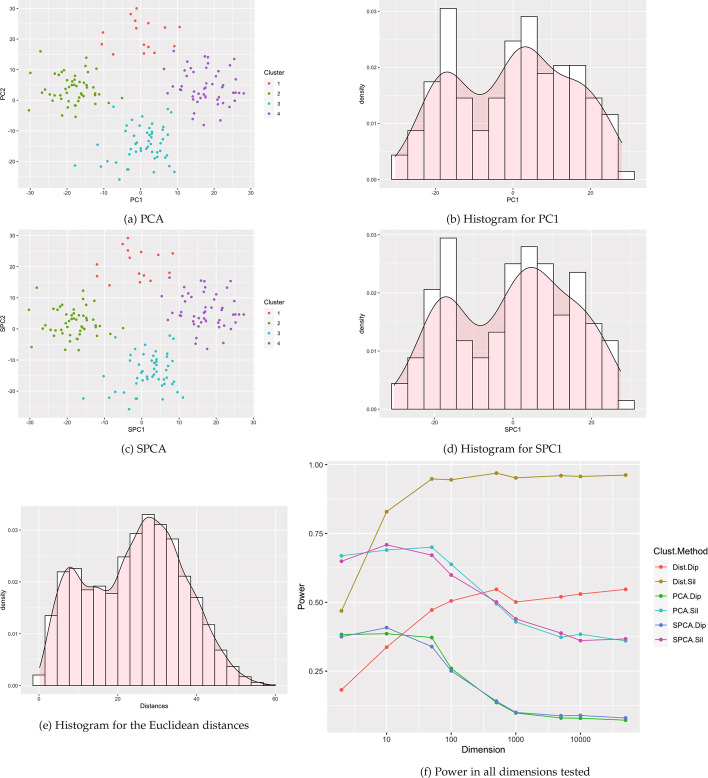
Visualizations for example Case 5: four clusters with one small cluster. All visualizations represent the use of a single example simulation in dimension $p=500$ except for Power which is measured in dimensions from $p=2$ to 50 K via estimation based off 1000 simulated data sets on each dimension

**Fig. 12:**
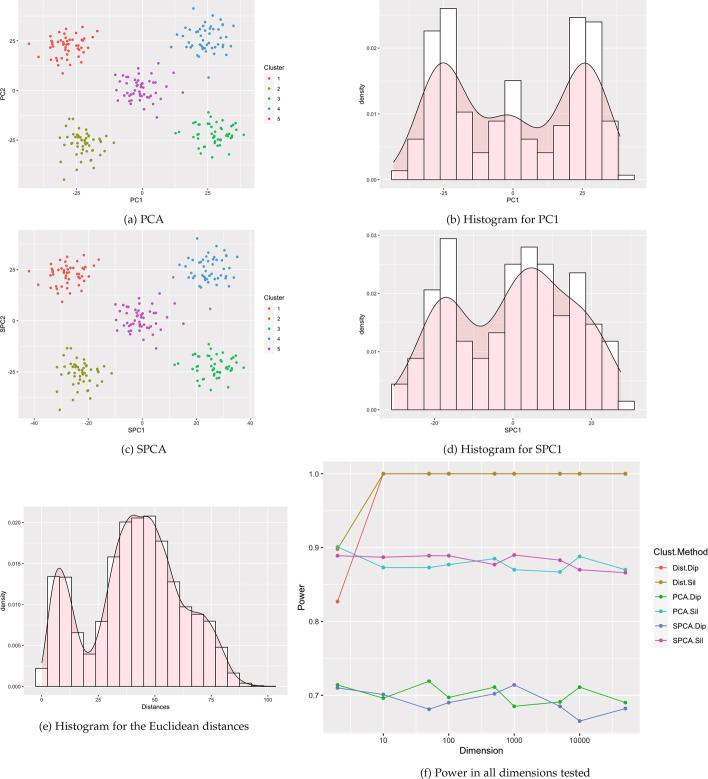
Visualizations for example Case 6: five clusters with one central cluster. All visualizations represent the use of a single example simulation in dimension $p=500$ except for Power which is measured in dimensions from $p=2$ to 50 K via estimation based off 1000 simulated data sets on each dimension

**Fig. 13:**
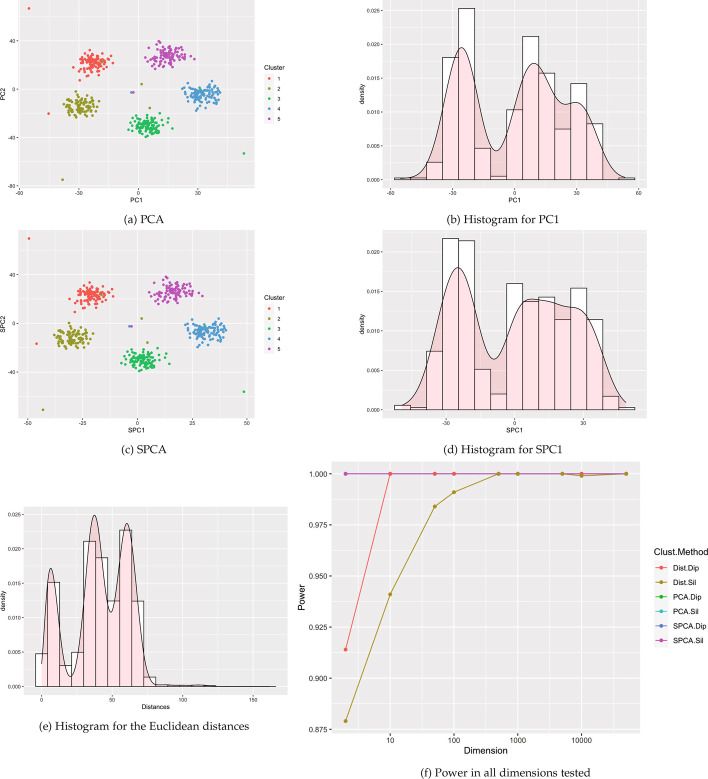
Visualizations for example Case 7: five clusters with ten outliers. All visualizations represent the use of a single example simulation in dimension $p=500$ except for Power which is measured in dimensions from $p=2$ to 50K via estimation based off 1000 simulated data sets on each dimension

**Fig. 14:**
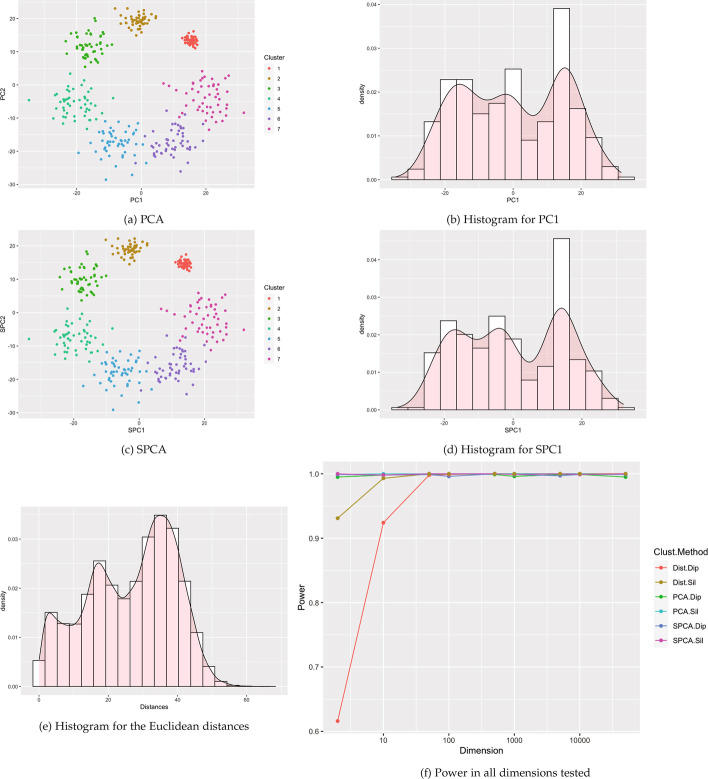
Visualizations for example Case 8: seven clusters with different variances. All visualizations represent the use of a single example simulation in dimension $p=500$ except for Power which is measured in dimensions from $p=2$ to 50 K via estimation based off 1000 simulated data sets on each dimension

**Fig. 15:**
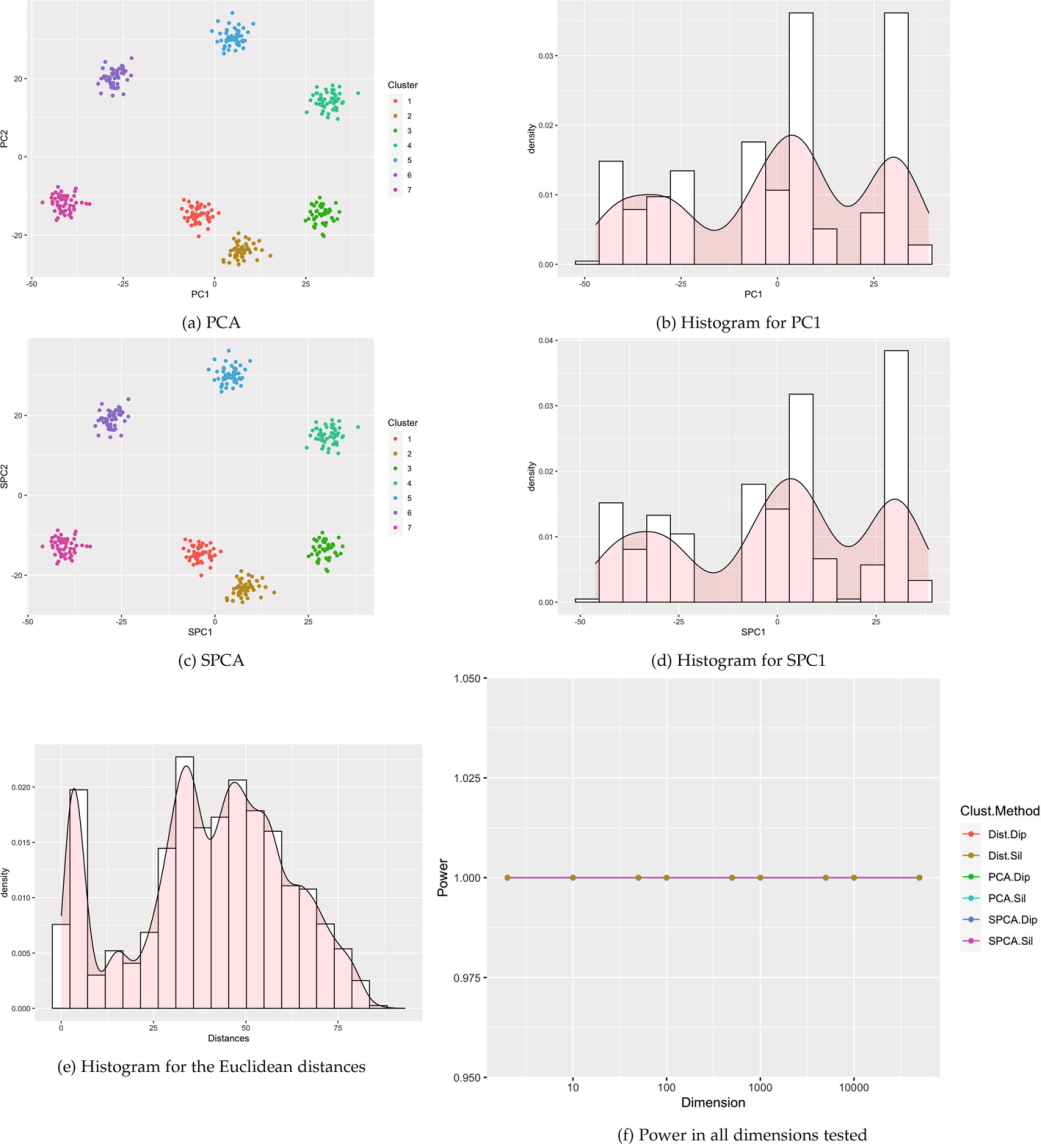
Visualizations for example Case 9: seven clusters with different push apart degrees. All visualizations represent the use of a single example simulation in dimension $p=500$ except for Power which is measured in dimensions from $p=2$ to 50 K via estimation based off 1000 simulated data sets on each dimension

**Fig. 16:**
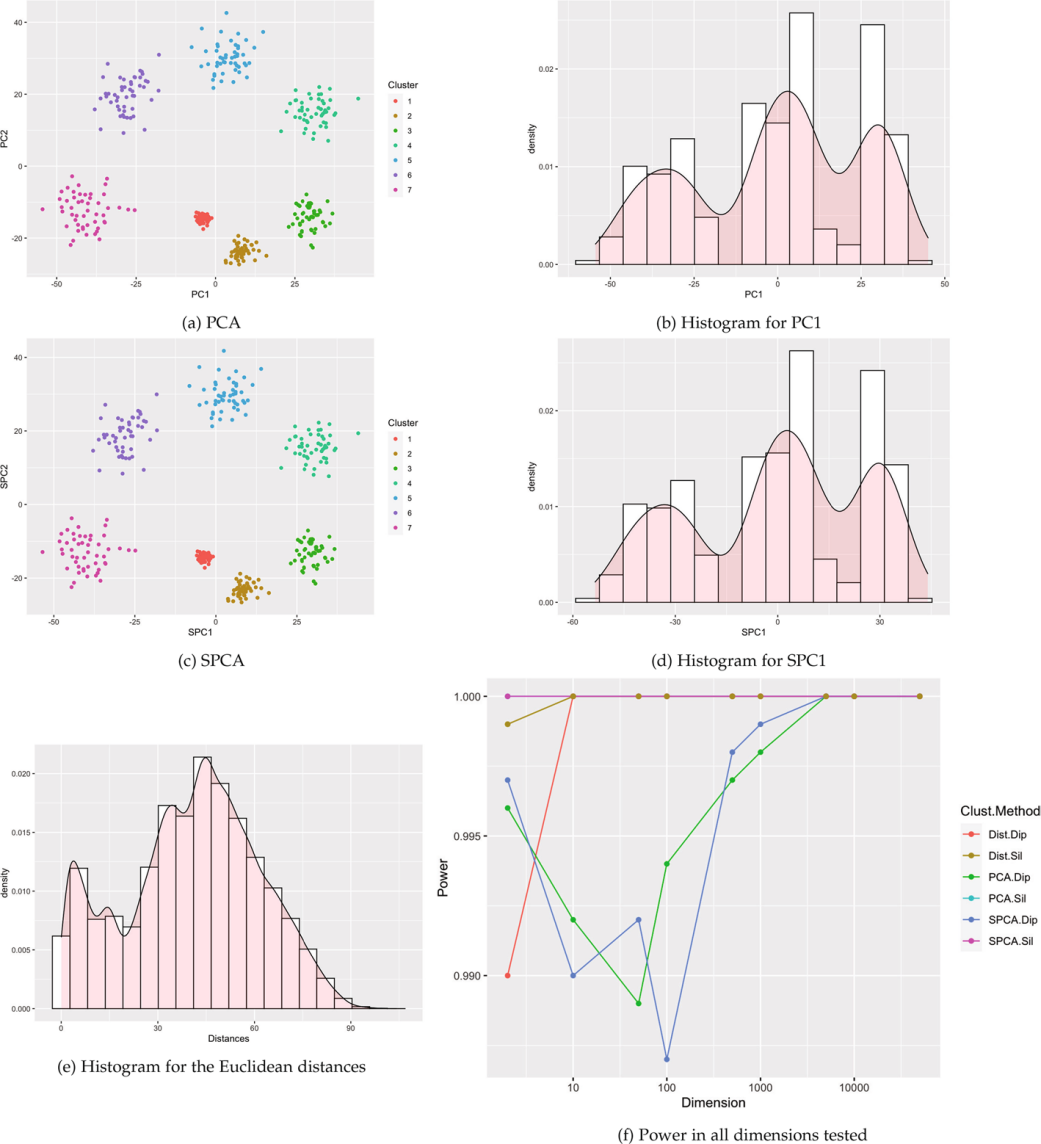
Visualizations for example Case 10: seven clusters with different push apart degrees and variances. All visualizations represent the use of a single example simulation in dimension $p=500$ except for Power which is measured in dimensions from $p=2$ to 50K via estimation based off 1000 simulated data sets on each dimension

#### Pan-cancer RNA-Seq

Figure 5 shows a pan-cancer analysis with 20,264 genes in 801 tumors across 5 tumor types contained in The Cancer Genome Atlas (TCGA) retrieved from the UCI Machine Learning Repository [16]. Distinct clusters are visible with the Kidney Renal Clear Cell Carcinoma (KIRC) separated from the rest of the groups in both PC1 and SPC1.

The first sparse principal component was deemed multimodal by both tests ($P = 1.32\times 10^{-6}$ for dip and $P = 0$ for Silverman^2^). For PC1, Silverman’s test on PC1 rejected the null hypothesis of no distinct clusters ($P = 0$), while the p-value for Hartigan’s dip test was just above the nominal value for PC1 ($P =.0512$). In contrast, the histogram for Euclidean distances appears unimodal, and tests of values from the Euclidean distance matrix were not statistically significant ($P \approx 1$ and $P =.622$ respectively).

#### Pan-lung cancer gene expression microarray

We then analyzed 54,675 gene expression probes from a dataset containing 293 lung tumor samples and 14 non-tumoral lung samples [18]. Samples were classified by sex, which was identified by plotting by plotting expression related to the Y chromosome (DDX3Y, using 205000_at) vs. expression related to X chromosome(s) (XIST, using 221728_x_at). Three male samples (GSM748078, GSM748189, GSM1163210) exhibiting female gene expression were excluded from further analyses. The remaining 304 lung tumor and non-tumoral samples [18] were normalized using iterative rank-based normalization [19] and log_2_ transformed.

In Fig. 6, the legend indicates that the data includes ten groups, with nine disease groups and one non-tumor group. SPCA and PCA show a large cluster consisting of several lung cancer groups, with the non-tumor group concentrated in the lower left portion of the graph, as well as a separate smaller cluster of the carcinoid group in the lower right. In the histograms of the first sparse principal component and the first principal component, the cluster structure is subtle, with the small cluster visible as slightly elevated in the upper tails. Previous research indicates that small, outlying clusters are often considered as separate clusters by Silverman’s test and considered as noise by the dip test. Histograms in Fig. 6 show multiple modes for PCA and SPCA but only one mode for distances. Indeed, when data was reduced by sparse PCA, Silverman’s test rejected the null hypothesis ($P = 0.0123$), while the dip test ($P = 0.991$). Similarly for PCA, Hartigans’ dip test did not detect cluster structure ($P =.989$), while Silverman’s test did ($P = 5.66\times 10^{-4}$). Values from the Euclidean distance matrix were not significant ($P =.999$ and $P =.41$, respectively, for dip and Silverman).

#### Squamous cell lung cancer shotgun proteomics

A proteomic dataset by [20] includes 4880 protein group measurements in 108 squamous cell lung cancer tissues across three groups, Inflamed, Redox, and Mixed. We used a subset of 3820 protein groups with no missingness. Intensity values, a measure of expression, were already log_2_ transformed and normalized by the authors (Fig. 7).

In all visualizations, the groups appear closely intertwined, with clusters in SPC1 and PC1 not well separated and all histograms appearing approximately unimodal. Statistically, both Hartigan’s dip test (with argument *mod0 = 1*) for unimodality ($P =.308$) and Silverman’s critical bandwidth test ($P =.216$) of all values from SPCA PC1 fail to reject the null hypothesis, likely due to the proximity of the Inflamed and Redox clusters. Other reduction-based tests of the cluster structure were similarly null ($P =.849$ and $P =.41$, respectively for PC1, and dip $P =.995$, Silverman $P =.222$ for Euclidean distances).

### Glioblastoma RNA-Seq

We next used a data set containing 1750 genes in 50 glioblastoma tumors [21] originating from The Cancer Genome Atlas (TCGA) and included with the M3C R package [22]. RNA-seq count data was previously processed and normalized; observations were previously assigned to one of four clusters, although a few observations were unassigned [21, 22]. Clusters are spread across SPC1 and PC1 (Fig. 8). Cluster structure was undetectable by all six tests.

## Discussion and future work

Traditional cluster analyses produce clusterings without testing whether their data was generated from distinct clusters rather than a single cluster. A lack of inherent distinct clusters could render cluster analysis inappropriate [1]. This paper provides and examines clusterability tests for analysis of high dimensional data, such as genomics, proteomics, and other data common in biomedical research. Without clusterability tests, researchers are unable to test for the appropriateness of cluster analysis, and unnecessary cluster analyses (including their results, which may inform future research) will persist throughout the biomedical literature. Importantly, clusterability tests are distinct from validation measures in that they do not require the user to first choose a specific clustering algorithm. (By contrast, silhouette coefficients and other post-clustering validation measures provide information on how well the results of a chosen clustering method fit the data.) For more details on this important distinction, please see our previous work [1].

The methods proposed in the present paper provide computationally efficient, valid tests for cluster structure before clustering data with a high number of features. The novel methods each conduct a multimodality test on the first sparse principal component of the data. Sparse PCA capitalizes on elastic net to select the most important features and produce principal components with sparse loadings, meaning only a small number of the loadings are nonzero. Sparse loadings can be more easily interpreted for real data when the number of features is large, such as for a genomic dataset with over 10,000 genes. Clusterability tests with the Silverman critical bandwidth test combined with SPCA or PCA detected known clusters in the single-cell, Pan-cancer RNA sequencing, and pan-lung microarray datasets and performed well in simulations. By contrast, reducing real-world high dimensional datasets to the sets of Euclidean distances rendered many known clusters undetectable.

As in all research, simulation studies are limited in scope. In this case, simulations were based on Gaussian data projected into high dimensional space via the clusterlab package [7]. Other simulations of high dimensional clusters structured differently could be tested in future work. Future studies could also investigate deeper relationships between the number of clusters and dimensions. Computational timing may vary depending on the computing infrastructure available to the user at the time of analysis.

To our knowledge, this project provides the first application of clusterability tests to real-world high dimensional data, including gene expression from proteomic microarray, bulk RNA-seq, single-cell RNA, and protein expression from shotgun proteomics. Additionally, it would be desirable to empirically test the performance of clusterability methods on realistic high dimensional datasets lacking cluster structure. Although we searched for such data, we were unable to find any, and we leave that task for future work. Similarly, we look forward to subsequent studies of more empirical data covering a wider range of parameters (e.g. additional exploration of *n*, *p*, and *c*).

Clusterability tests are not infallible. All methods tested thus far lack power for when clusters are very close in space. Separate ongoing theoretical research (results not shown) shows that these conclusions often follow naturally from the mathematics of the underlying distributions of the data. Thus, future theoretical study could explore mathematical properties suggesting how or if clusterability tests could increase power for such data. Development of enhanced clusterability testing in the presence of well-integrated clusters is an open problem.

Our methodology depends on a unidimensional reduction, which may result in a loss of information in the dataset that is more apparent in more than one dimension, such as in the first two principal components. At the time of writing, reducing the data to a single dimension, such as by one of the methods in the paper, is required to run the multimodality tests, and thus to run the clusterability tests themselves. Alternative methods of dimension reduction, alternative distance metrics (e.g. correlations between profiles) and further development of multimodality tests in higher dimensions are open areas for future research.

## Conclusions

In summary, we provide the methodology, simulations, empirical analyses, and user-friendly algorithms to encourage broad future use of clusterability tests on high dimensional data. Methods with the dip test and either sparse PCA or traditional PCA detected known cluster structure in high dimensional-omics based cancer data and had high power in simulations. Type I error was controlled at or below the nominal level across all dimensions for all methods. Consequently, we recommend the utilizing clusterability tests for high dimensional data based on SPC1 or PC1. The decision of whether to use the dip test or Silverman’s test depends on the scientific importance of small clusters in the user’s field of application. We hope that these methods and clusterability tests in general become more common in the practice of cluster analysis.

## Methods for clusterability analysis

Our paper describes a total of 6 methods to test for clusterability by combining 2 tests for unimodality with 3 reduction methods, shown in Fig. 3. Multimodality methods are described in the subsection below on Multimodality tests Reduction methods are detailed in the subsection below on Dimension Reduction Methods. Two new methods are proposed in this paper utilizing a new dimension reduction method for use with high-dimensional data. These proposed methods are shown in Fig. 3 in the top middle yellow bubble and orange bubbles on the right. The next four methods were proposed previously [1] but restricted to testing on small datasets. These are denoted with the combination of purple and green bubbles. The bottom red arrow, included for completeness, is for single dimensional data. In this paper, all methods are tested for simulated and empirical data with varying numbers of observations and features, as described in the Results section.

### Multimodality tests

Clusterability tests involve statistical tests of multimodality on the uni-dimensional reduced dataset, with reductions described in the subsection, Dimension Reduction Methods. Intuitively, multimodal distributions correspond to data with cluster structure and unimodal distributions correspond to more homogeneous data. Two multimodality tests are described: Hartigan’s Dip test and Silverman’s Critical Bandwidth test. Both tests define a null hypothesis that the data is generated from a specific class of unimodal distributions. If the observed data deviates enough from that prior assumption, then the user may reject the null hypothesis. The alternative hypothesis is that the data is generated from a multimodal distribution.

#### Hartigan’s Dip test

We reproduce the notation in [23] to define the Dip statistic. First, define $\rho (F,G)$ as the maximum difference between the empirical distribution function *F* and another bounded function *G*

1 $$\begin{aligned} \rho (F,G) = \sup _{x \in \mathcal {X}}{|F(x) - \mathcal {G}(x)|} \end{aligned}$$

Next, take the minimum value of over all the class of unimodal distribution functions $\mathcal {U}$

2 $$\begin{aligned} \rho (F,\mathcal {U}) = \inf _{G \in \mathcal {U}}{\rho (F,G)} \end{aligned}$$

The *dip* *D*(*F*) of a distribution function *F* is defined as “the maximum difference between the empirical distribution function and the unimodal distance function that minimizes the maximum difference.” That is:

3 $$\begin{aligned} D(F) = \rho (F,\mathcal {U})=\inf _{G \in \mathcal {U}}{\rho (F,G)} = \inf _{G \in \mathcal {U}}\sup _{x \in \mathcal {X}}{|F(x) - \mathcal {G}(x)|}. \end{aligned}$$

*D*(*F*) measures departure from unimodality. The dip test rejects the assumption of unimodality if the dip is sufficiently large, indicating that the data differs from the closest uniform distribution. Computational details can be seen in Hartigan’s paper [23]. The diptest is available in an R package [24] for convenience. However, others have documented the excessively conservative nature of the dip test and recommended a calibration [11] that is not yet implemented in standard software as of the writing of this paper.

#### Silverman’s critical bandwidth test

Define the critical bandwith $h_{\text {crit}}$:

4 $$\begin{aligned} h_{\text {crit}} = \inf \{h: \hat{f}(\cdot , h)\text { has at most one mode}\} \end{aligned}$$

where,

5 $$\begin{aligned} \hat{f}(t, h) = \frac{1}{nh}\sum _{i = 1}^{n}K\left( \frac{1}{h} (t - X_i)\right) \end{aligned}$$

and $X_1 \dots X_n$ denotes data with *n* observations, and *K* is the density of the standard normal distribution. Here $\hat{f}$ is the *kernel* estimate of a density (distribution) function *f*. The Silverman test [25] rejects the unimodality assumption if *f* requires a sufficiently large $h_{\text {crit}}$ to produce an empirical distribution based on one Gaussian component rather than a Gaussian mixture. An R package is available [26] for this calculation, including a recommended calibration [27].

### Dimension reduction methods

Denote the full set of data in the matrix $\varvec{X}=(X_1^\prime ,\ldots ,X_n^\prime )^\prime$ with *n* observations and *p* features, where $X_i=(X_{i1},\ldots ,X_{ip})^\prime$ is the vector of *p* features for observation $i=1,\ldots ,n$. Given that the multimodality tests in the Multimodality Tests section are not guaranteed to work properly in a multiple dimensions [1] (i.e. when $p>1$), we need to perform a reduction to a single dimension of data before the tests are applied. Below we list the methods we have used.

#### Distances

Compute the distance matrix between all of the pairs of vectors. In this manuscript, we have used Euclidean distances. However other distance metrics may be considered. The resulting upper or lower triangle of the distance matrix is flattened into a vector of distances to which we apply the multimodality test.

#### PCA

Principal component analysis (PCA) is a common, well-studied dimension reduction technique [28, 29]. We take the first principal component (PC1) and use this uni-dimensional distribution to apply the multimodality test.

#### SPCA

Sparse PCA (SPCA) [4], which uses an elastic net objective function to produce sparse loadings, has experienced increasing popularity since its publication in 2006. We hypothesize that SPCA may perform better and be more interpretable than standard PCA for large datasets. We extract the first sparse principal component (SPC1) and use this uni-dimensional distribution to apply the subsequent multimodality test.

## Data Availability

Simulation code for this project is available on a gitlab repository at https://gitlab.moffitt.usf.edu:8000/Bios2Projects/brownstein_naomi/2103_clusterability_2019. Data were acquired from publicly available sources. Specifically, the single-cell data is available at https://support.10xgenomics.com/single-cell-gene-expression/datasets/3.0.0/pbmc_10k_protein_v3. TCGA pan-cancer data is a collection of ML datasets downloadable from https://archive.ics.uci.edu/ml/datasets/gene+expression+cancer+RNA-Seq. Pan lung cancer data is available at https://www.ncbi.nlm.nih.gov/geo/query/acc.cgi?acc=GSE30219. Squamous cell lung cancer proteogenomics may be found in the Supplemental Data 3 (41467_2019_11452_MOESM6_ESM.xlsx) from https://pubmed.ncbi.nlm.nih.gov/31395880/. Glioblastoma data can be downloaded within the M3C library in R, available from https://www.bioconductor.org/packages/release/bioc/html/M3C.html.

## Appendix

### Detailed summary of power simulations

- Case 2 (4 clusters, equal variance—Fig. 2): Across all dimensions, power for methods combining Silverman with PCA or SPCA were similar (around 60%) and higher than the corresponding dip tests (around 40%), which were also higher than running the dip test on Euclidean distances (below 30%). Silverman’s test on the distances was less powerful then Silverman’s test on sparse PCA or standard PCA for low dimensions and more powerful for higher dimensions (above 75% for 50+ dimensions).
- Case 3 included 4 clusters, 2 of which have different variances (Fig. 9). While all methods had high power (>95%), distance methods had the highest power (100%) in 10 or more dimensions, dip combined with PCA or SPCA at 98–99%, and Silverman with PCA or SPCA at around 95–97%. There was no clear relationship between power and dimension.
- Case 4 was similar to Case 2 with equal variances except one of the clusters was farther away from the other 3 (Fig. 10). Power for all methods increased with increasing dimension. In two dimensions, Silverman with either component (SPC1 or PC1) was the most powerful, then dip with either component or distance Silverman, and lastly distance dip. For 10 or more dimensions, distances with either test had greater power than Silverman with PCA or SPCA followed by greater power than dip with PCA or SPCA; in each case, power for SPCA was slightly higher than for PCA. For 500 or more dimensions, power converged at or near 100% for all methods.
- Case 5: 4 clusters with 1 smaller than the others (Fig. 11). Power decreased across dimension for reductions with PCA and SPCA and increased across dimensions for distances. Thus, the ranking of methods dependent on dimension. In two dimensions, Silverman with SPCA and PCA were most powerful, followed by Silverman’s test on the distances, dip with PCA or SPCA, and finally dip with distances. In ten dimensions, Silverman’s test on the distances was most powerful, followed by Silverman with SPCA and PCA, and all dip tests, with distance dip the lowest. In 50 and 100 dimensions, distance dip overtook the dip test on SPCA or PCA, but these methods remained lower than all methods using Silverman’s test. For dimensions of at least 500, distance methods showed the highest power, then Silverman with PCA or SPCA, and then dip with PCA or SPCA.
- Case 6: 5 clusters, with 4 clusters forming a rectangle and 1 cluster in the center (Fig. 12). Distance methods were most powerful (100%) for all but 2 dimensions. PCA and SPCA were comparable and constant across dimensions, with higher power for Silverman than dip (nearly 90% compared to about 70%). In two dimensions, all Silverman methods were comparable, with distance dip slightly lower, and dip combined with PCA and SPCA the lowest.
- Case 7: 5 clusters, arranged approximately in a circle with a few outliers scattered about (Fig. 13). Power was 100% for all dimensions for methods using SPCA and PCA. The two methods reducing via distance exceeded 85% for two-dimensions and increased across all dimensions, with power for dip exceeding that of Silverman. Power reached 100% for distance dip and distance Silverman at 10 dimensions and 500 dimensions respectively.
- Case 8 had 7 clusters with different variances arranged in a circle (Fig. 14). All methods had nearly perfect power for at least 50 dimensions, and only distance based methods had lower power for lower dimensions, with Silverman exceeding dip. Distances with Silverman had over 90% power across all simulations, while distances with dip only had about 60% power in 2 dimensions.
- Case 9 had 7 well separated clusters with equal variances in a different orientation (Fig 15), and all methods had 100% power in all dimensions.
- Case 10 had the orientation of Case 9 with different variances (Fig. 16), with all methods displaying high power exceeding 98% and PCA and SPCA Silverman having consistently perfect power. Distances had 100% power for at least 10 dimensions, with Silverman exceeding dip in 2D and distance dip the lowest performing method in 2D. PCA and SPCA combined with dip were slightly lower, with power decreasing slightly across low dimensions (10 or 50) before increasing at $p=100$.

## Footnotes

1 The clusterlab package has been archived since the simulations were completed. We retested the package and did not find any defects when optional plots were supressed. We hypothesize that this archival is due to lack of responsiveness, maintainance and improvement of the clusterlab package upon external updates of ggplot2, rather than to any problems with the cluster generation process.

2 The Silverman test can round p-values below 0.005 to zero, as discussed previously [17].
